# Enhancing diversity in public health scholarship: the role of publication mentorship

**DOI:** 10.1093/heapol/czaa104

**Published:** 2020-11-09

**Authors:** Dena Javadi, Sameera Hussain

**Affiliations:** c1 World Health Organization; c2 Public Health Agency of Canada, 130 Colonnade Road S, Ottawa, ON K1A 0K9, Canada

**Keywords:** Mentorship, women, equity, research, publication, scholarship, leadership

** KEY MESSAGES**

- Women and other groups facing structural barriers are underrepresented in research, scholarship and leadership.
- Culturally responsive mentorship can serve as part of a solution in strengthening capacity and enhancing social capital in academic and research settings.
- The increased diversity of perspectives and thought afforded by improved representation strengthens public health scholarship and its contributions to social justice.

## Introduction

This Supplement, a collaboration between Health Systems Global (HSG) and the Alliance for Health Policy and Systems Research (AHPSR) and *Health Policy and Planning* (HPP), is the product of a six-month publication mentorship programme aimed at supporting early-career women conducting Health Policy and Systems Research (HPSR) in low- and middle-income countries (LMICs) (AHPSR, 2019). The mentorship programme guided mentees—selected based on a motivation letter and draft abstract—in preparing a high-quality manuscript for a peer-reviewed journal. Topics for submitted papers were restricted to themes identified for the Sixth Global Symposium on Health Systems Research on ‘Re-imagining health systems for better health and social justice’ (HSR, 2020).

This editorial will discuss the role of programmes such as publication mentorship in mitigating structural barriers in the career advancement of women, racial and ethnic minorities and others experiencing discrimination and bias. It will also highlight the social justice and equity-oriented themes that connect the papers included in the special issue, demonstrating the importance of amplifying voices that are often underrepresented in scholarship and leadership.

## Women in academia

It is well-documented that gender, race, ethnicity, socioeconomic status and other combinations of social identities impede women’s progress in the health workforce and restrict access to corridors of power (Hankivsky *et al.*, 2014; Newman *et al.*, 2017; Clark *et al.*, 2018; Moyer *et al.*, 2018; George *et al.*, 2019; WHO, 2019; Yount *et al.*, 2020). For example, while women represent 53% of the world's bachelor's and master's degree graduates and 43% of PhD graduates, they represent only 28% of researchers in all fields, suggesting a high rate of attrition in the academic workforce, limiting diversity of perspective and thus scholarship (Tiedeu *et al.*, 2019).

Women’s progress in academic medicine and public health is often impeded by structural barriers and gendered institutions (Edmunds *et al.*, 2016; Howe-Walsh and Turnbull, 2016; Stavroula *et al.*, 2017; Yousaf and Schmiede, 2017; Cameron *et al.*, 2019). Some of the key barriers include gendered stereotypes and bias, imbalanced family and care-taking roles, lack of mentorship and poor organizational support (Edmunds *et al.*, 2016; Corneille *et al.*, 2019; Shreffler *et al.*, 2019). Institutions have begun to implement practices aimed at improving the recruitment, promotion and retention of women and others facing structural barriers. These include institution-wide training in unconscious bias, career development support, childcare services, availability of culturally responsive mentorship, networking opportunities, diversity practices in recruitment and promotion committees and others (Carr *et al.*, 2017).

In academia, high-impact publications continue to serve as a benchmark of productivity, expertise and success. Therefore, gender disparities in the number of publications, especially amongst early- and mid-career researchers, contribute to inequalities in academic placements and promotions (Mueller *et al.*, 2016; Cooper *et al.*, 2019). This is observable in the rates of assistant versus full professorships across gender and race categories, with fewer women holding full professorships as compared with men, and minority women underrepresented at all levels (Patel *et al.*, 2018). An intersectional lens—accounting for interactions across gender, race, ethnicity, disability and other social identities—is required to improve measures designed to empower those facing structural barriers.

## Mentorship as part of the solution

Culturally responsive mentorship is a powerful tool in strengthening capacity and enhancing social capital (Sopher *et al.*, 2015; Campos, 2016; Bertrand Jones *et al.*, 2020). It is important to note that mentorship is not a flawless panacea. For example, without formal and informal structures to ensure equity in mentorship programmes, mentorship roles—seen as ‘nurturing’—tend to fall disproportionately to women (Campos, 2016). Furthermore, mentoring relationships are strengthened when connected to wider networks creating cultural shifts in homogenous institutions (DeCastro *et al.*, 2013). Mentorship must be viewed as a two-way relationship whereby the perspectives and experiences of the mentee are treated with respect, leading to meaningful dialogue and debate around culturally responsive tools, approaches and methods in conducting and disseminating research. Mentoring arrangements not only strengthen the capacity of mentees in navigating established institutions and protocols, but also bring new thinking and perspectives to mentors, thereby enhancing the adaptive capacity of the scholarship.

## Increasing diversity of thought in scholarship

HPSR is a dynamic field focused on strengthening resilient health systems, addressing systemic barriers, enhancing social justice and leaving no one behind. Therefore, all measures that diversify the field and amplify underrepresented voices can help to enrich its scholarship with new perspectives, approaches and methods. In their commentary on ‘Supporting early-career mentorship for women in HPSR: a vital input to building the field’, Kwamie and Jalaghonia expand on the significance of mentorship for HPSR and provide insight based on lessons learned from the Alliance-HSG publication mentorship programme. They reflect on positive outcomes from the programme, such as professional development opportunities for both mentors and mentees, as well as limitations, such as short timelines and language barriers.

The papers included in this special issue include a wide range of equity-oriented topics affecting health system performance and outcomes. These include intersectoral and systemic barriers and facilitators affecting individual-level behaviours, health system structures affecting equity in service delivery, as well as institutions and policies inhibiting representation and inclusion.

## Barriers and facilitators affecting individual behaviours

Tran *et al.* identify links between poor reproductive health outcomes and Intimate Partner Violence (IPV) in Bangladesh, highlighting the role of education and food security in mitigating these outcomes. Luhumyo *et al.* examine IPV prevalence in Western Kenya and its links to prematurity as a perinatal outcome among survivors, also highlighting the role of education and formal employment. Nanyonga *et al.* examine barriers and facilitators for nurses in leadership, demonstrating the importance of organizational culture in enhancing nurses’ ability to lead and engage in policy decisions. Faith *et al.* assess utilization of inactivated polio vaccination among caregivers with children in Uganda, identifying a need for social mobilization and outreach to improve vaccination coverage.

## Health system structures affecting equitable service delivery

Sunkwa-Mills *et al.* consider the role of peer support in neonatal intensive care units in Ghana, emphasizing a gap in the hospital system partially met by informal support structures. Ekenna *et al.* look at implementation factors in a free maternal and child health programme in Nigeria, citing corruption, lack of political support and financial barriers as reasons for discontinued access to free care. Omer *et al.* assess effectiveness of nutritional training on health providers’ counselling of pregnant women in Ethiopia, finding improved outcomes and better alignment with international recommendations.

## Institutions and policies affecting representation and inclusion

Naher *et al.* study community-led transparency and accountability measures to combat health sector corruption in South and South-East Asia, featuring social audits, resource decentralization, e-governance, online transparency platforms and community scorecards as examples of effective strategies. De Moura Pontes *et al.* consider what is needed to achieve Universal Health Coverage in Brazil, with a focus on indigenous populations, stressing the importance of establishing culturally relevant approaches and ensuring inclusion in the formulation and execution of health actions.

## Conclusion

The papers featured in this special issue are a product of collaboration and mentoring relationships between early-career researchers and those established in HPSR, and the editorial team at HPP. The range of topics and contexts they cover demonstrates the value of diversity in research and scholarship.

The COVID-19 pandemic has further highlighted disparities in research output experienced by women (Malisch *et al.*, 2020). This is in part due to the unintended consequences of public health measures implemented worldwide, leading to exacerbated disparities in gender roles and caregiving obligations, compounded by the lack of social support systems. Academic institutions, donors and journals all have a role to play in addressing these inequities and enriching global scholarship through creating more inclusive academic and research spaces.
